# Motor Imagery-Based Rehabilitation: Potential Neural Correlates and Clinical Application for Functional Recovery of Motor Deficits after Stroke

**DOI:** 10.14336/AD.2016.1012

**Published:** 2017-05-02

**Authors:** Yanna Tong, John T. Pendy, William A. Li, Huishan Du, Tong Zhang, Xiaokun Geng, Yuchuan Ding

**Affiliations:** ^1^China-America Institute of Neuroscience, Luhe Hospital, Capital Medical University, Beijing, China; ^2^Department of Neurology, Luhe Hospital, Capital Medical University, Beijing, China; ^3^Department of Neurosurgery, Wayne State University School of Medicine, Detroit, Michigan, USA; ^4^China Rehabilitation Research Center, Capital Medical University, Beijing, China

**Keywords:** movement, muscle activation, motor function, neural correlates, clinical implication

## Abstract

Motor imagery (MI), defined as the mental implementation of an action in the absence of movement or muscle activation, is a rehabilitation technique that offers a means to replace or restore lost motor function in stroke patients when used in conjunction with conventional physiotherapy procedures. This article briefly reviews the concepts and neural correlates of MI in order to promote improved understanding, as well as to enhance the clinical utility of MI-based rehabilitation regimens. We specifically highlight the role of the cerebellum and basal ganglia, premotor, supplementary motor, and prefrontal areas, primary motor cortex, and parietal cortex. Additionally, we examine the recent literature related to MI and its potential as a therapeutic technique in both upper and lower limb stroke rehabilitation.

Degenerative processes are the primary underlying cause of several conditions generally associated with aging, including cardiovascular disease, stroke, and cancer [1]. Of these conditions, stroke remains one of the major worldwide causes of mortality, and is a leading cause of serious long-term disability in the United States [2]. Even after completing standard rehabilitation regimens, 50-60% of stroke patients suffer from some measure of motor impairment [3]. Of this population, older stroke patients are often substantially impaired in their ability to perform activities of daily living, thus necessitating long-term rehabilitation services [4]. It is therefore crucial to examine novel post-stroke therapeutic techniques in order to facilitate effective stroke recovery. Constraint-induced movement therapy (CIMT) is a popular rehabilitation strategy for many stroke patients, and forces the use of affected muscles by restricting the use of unaffected limbs [5]. However, severely injured patients are often devoid of even residual movement in affected limbs; thus, CIMT and other movement-based therapies cannot be employed to assist these patients. This limitation has compelled the scientific community to explore other therapeutic techniques, including motor imagery (MI) training [6].

MI is a popular research focus in the fields of neurophysiology, neuroimaging, neurology, and psychology. Additionally, it has been developed as a foundation for neurorehabilitation and brain-machine and brain-computer interfaces [7]. However, a detailed description of MI and its application in stroke rehabilitation is lacking. Therefore, the aim of this review is to identify and examine the roles of neural correlates underlying MI, and to examine the latest literature associated with MI-based stroke rehabilitation.

## Concept of Motor Imagery

Presumably, the signal flow in a motor control system can be divided into four stages: 1) a motor signal is generated in the motor cortex, 2) the motor command travels through the spinal cord, 3) the motor command activates specific muscles, and 4) conscious and unconscious sensory feedback is transmitted back to the brain after muscle contraction, terminating in somatosensory cortex. This flow of information constitutes the sensory-motor closed loop. In the planning stage of motor control, information regarding the potential movement is acquired, but explicit specification of motor parameters is excluded [10]. The motor command is preceded by a preparation stage, and the organism waits for an execution cue before movement is permitted [10]. MI corresponds to activation of neural representations of potential movement, and is considered functionally equivalent to the planning and preparation stages (though *not* the execution stage) of motor control [7, 11, 12]. This suggests that MI and motor execution are generated through analogous computational steps [13], and involve similar brain structures. Indeed, imagining a simple, highly automatic movement often takes a similar amount of time as compared to the amount of time necessary to execute that same movement, though more variation exists for increasingly complex movements [14].

## Neural Correlates of MI and Clinical Implications

MI is a promising neurorehabilitation technique, particularly for stroke rehabilitation, because it appears to involve the control mechanisms and neural substrates employed in actual movement [8]. Researchers have been exploring the brain structures involved in MI for over two decades, and although the precise neural correlates remain unclear, much progress has been made [7]. A plethora of neuroimaging studies have demonstrated that the cortical and subcortical regions activated during MI tasks substantially overlap with those involved in movement execution. In the subsequent sections, we will briefly review the neural correlates of MI and, when possible, the impact of stroke on these brain structures and subjects’ abilities to perform MI.

### Cerebellum and Basal Ganglia

Generally, portions of the cerebral cortex considered to be involved with motor control include the primary motor cortex (M1), the supplementary motor area (SMA), and the premotor cortex (PMC). These cortical areas are closely linked to the cerebellum and basal ganglia, resulting in extensive feedback loop systems [15]. These loop systems permit the coordination, cortical modulation, and feedback control that have been considered the primary functions of the cerebellum [16, 17], yet until recently, the manner in which the cerebellum influences MI tasks was unclear. A 2016 study using cerebellar transcranial direct current stimulation demonstrated that the cerebellum has an inhibitory effect on MI, and functions by preventing efferent impulses (induced by MI) from reaching medullary and skeletal muscular levels [18]. As it relates to stroke, few studies have directly examined the impact of cerebellar lesions on MI abilities. In a small transcranial magnetic stimulation study of eight patients with unilateral cerebellar lesions due to thromboembolic stroke, ischemia in the posterior inferior cerebellar artery territory decreased the excitability of motor cortex, and resulted in diminished MI abilities as compared to a group of aged-matched, healthy controls [19]. An earlier study of cerebellar stroke substantiated these findings [20].

In addition to cerebellar activation, MI has been shown to recruit subcortical motor areas such as the basal ganglia [21]. It has long been known that patients with Parkinson’s disease demonstrate reduced ability to perform MI tasks [22-24]. Although the substantia nigra damage seen in Parkinson’s patients is not functionally equivalent to stroke-induced basal ganglia damage, these studies highlight the importance of the basal ganglia in MI-based tasks [25]. Additionally, in a study of 37 hemiplegic stroke patients, there was a correlation between presence of putamen damage and diminished MI capacity [26]. Further, a recent systematic review indicated that basal ganglia damage, specifically to the putamen, may negatively impact MI abilities [27].

### Premotor, Supplementary Motor, and Prefrontal Areas

MI-induced brain activity typically involves premotor and supplementary motor areas, the two brain regions most consistently implicated in MI processes [7]. Indeed, a recent study on MI and motor execution in stroke patients confirmed the activation of the PMC and SMA in MI and motor execution in a control population [28]. Additionally, previous studies have indicated that premotor and supplementary motor areas play a key role in the planning, preparation, and control of movement [10, 28, 29]. Some investigators have found that locations of SMA activity in MI only partially overlap with those of motor execution [30], implying that portions of the SMA may play a specific role in MI alone [31]. A study using magnetoencephalography (MEG) [32] suggests that some neurons of the SMA inhibit M1 activity, thereby preventing motor execution. This is in accordance with the results of an earlier study that demonstrated the suppressive effect of the SMA on M1 [33]. More recently, the influence of the SMA on M1 activity was examined in an effective connectivity analysis of the damaged (experimental) and undamaged (control) cerebral hemispheres of 10 stroke patients [34]. After MI- and motor execution- based tasks, the authors suggested a suppressive influence of SMA on M1 during MI, whereas the effect of SMA on M1 was unrestricted during motor execution.

Overlapping activity of brain regions during MI and motor execution has also been identified in the PMC [35]. Particularly, the dorsal portion of the PMC (PMCd) has been shown to be activated in MI [30], and has been posited as the underlying source of both MI and motor execution [34]. Though discrepancies have been reported regarding the role of more ventral portions of the PMC (PMCv), a 1995 study demonstrated activation of the PMCv during both MI and motor execution [30]. A 2014 study also confirmed activity in the left PMCv during MI of upper and lower extremities [36]. Additionally, studies on primates have shown that both the PMCd and the PMCv play a key role in the planning, preparation, *and* execution of motor actions [37]. Despite the overlap, regions of the PMC have been specifically implicated in processes likely related to MI. For example, the PMCd has been linked to impulse control, which is believed to limit premature motor response initiation and may be a key element in the divergence between MI and motor execution [38, 39]. Further, in a study on motor imagination in amputees, PMCd activation appeared more rostral in MI than in motor execution, suggesting that certain neurons of the PMC are related solely to MI tasks [40].

Frontal cognitive regions have also been examined in neuroimaging studies of MI that ultimately indicated various roles of prefrontal areas. Though prefrontal activity is typically considered in relation to cognitive processes [41], both the ventral prefrontal cortex and the anterior cingulate cortex have been associated with the inhibitory control of movement during the preparation stage of motor control [39, 42]. Additionally, several studies have employed functional magnetic resonance imaging (fMRI) to examine novel roles of prefrontal areas in MI tasks related to familiar and unfamiliar objects [43], imagined force generation [44], and eccentric and concentric muscle contraction [45]. These, among others, have confirmed the role of prefrontal areas in MI tasks. As it relates to stroke, prefrontal lesions have been shown to impact the vividness of MI [26]; however, the relationship between prefrontal cortex and MI should be further explored in stroke patients, in order to isolate the specific effects of prefrontal cognitive regions on MI-related processes.

### Primary Motor Cortex

Activation of M1 during MI has historically been more controversial in the literature than the well established activation of premotor areas [31], and typically, M1 activation during MI tasks has been considered minor in comparison to M1 activation during motor execution [46]. As mentioned previously, MI is functionally equivalent to motor preparation, which is typically succeeded by motor execution. The debate surrounding the significance of M1 to MI training was initially due to the conflicting results of several studies [7], some of which indicated M1 activation during MI tasks, and some of which did not. However, recent examinations have firmly supported the notion that M1 is activated during MI tasks [28, 47-50], though the direct role of M1 in facilitating muscle contraction is less clear. In patients with limited residual post-stroke motor function, MI activation of M1 may be beneficial for “relearning” motor patterns disrupted by stroke [51]. Indeed, Szameitat et al. concluded that due to its ability to activate M1, MI is the most promising approach to activating the motor system in hemiparetic stroke patients (when compared with passive movement and movement observation).

However, a small study of patients at least eight months removed from stroke demonstrated that MI does not appear to significantly activate the ipsilesional M1 (i.e. the stroke-damaged M1), indicating that the role of MI in directly facilitating motor output is limited [52]. Conversely, in a 2009 fMRI study of well-recovered stroke patients, Sharma et al. clearly identified M1 activation during an MI-based finger-thumb opposition task [50]. Although M1 was active during MI, the motor system remained disordered, often with bilateral M1 and PMCd involvement. The group therefore concluded that components upstream of M1 might be more effectively targeted by rehabilitation strategies, particularly in severely affected patients.

A more recent analysis of hemiplegic stroke patients examined the functional connectivity between ipsilesional M1 and the entire brain using resting state fMRI [53]. As expected, stroke altered functional connectivity and disrupted motor pathways (as compared to healthy controls). Though neural reorganization is not the topic of this review, Zhang et al. demonstrated augmented functional connectivity between ipsilesional and contralesional M1 and diminished functional connectivity between ipsilesional M1 and ipsilesional SMA (shown to be inhibitory to M1, discussed in Section 3.2) after MI training in addition to conventional rehabilitation. Despite the lack of an appropriate control population, the group uncovered a statistically significant correlation between functional connectivity changes and Fugl Meyer Assessment (FMA) score changes after MI training, suggesting a possible, though preliminary, benefit to MI. Clearly, further research is needed to specify the role of M1 activation in MI training for stroke rehabilitation.

### Parietal Cortex

The parietal cortex plays important roles in both sensory integration [9] and motor execution [54, 55], and evidence strongly suggests that the parietal cortex substantially influences MI. Though parietal regions are activated during both MI and movement execution, a 2000 fMRI study demonstrated that regions of parietal cortex are differentially activated during real and imagined hand movements [35]. In a more recent study, the posterior parietal cortex (PPC) was revealed to be more active during imagined movements than during motor execution [40]. Further, many neuroimaging studies have established the presence of MI-induced PPC activity, as well as MI deficits following PPC damage [7, 56]. Parietal regions such as the inferior parietal lobule [57], the supramarginal gyrus [58], and the superior parietal lobule [59] have also been implicated in MI tasks. Highlighting the necessity of intact parietal structures for vivid MI, a 2016 systematic review indicated that subjects with parietal lobe damage were the most substantially impaired in their ability to perform MI [27].

## MI for Post-Stroke Rehabilitation

### Upper Limb Training

Despite a substantial body of literature and knowledge, no neuroprotective treatments are currently employed to combat stroke [60]. Thus, we focus on MI as a promising neurorehabilitation technique for stroke patients, particularly considering the analogous pre-processing steps and structural overlap between MI and movement execution. Recent investigations have focused primarily on MI in conjunction with other types of therapies for optimal motor recovery, and most studies regarding MI-based neurorehabilitation have evaluated its efficacy in relearning tasks performed with the upper extremities. In a rehabilitation study of 10 stroke patients, MI was used in addition to both conventional physiotherapy and either synchronous (MISAO) or asynchronous action (MIAOO) observation for four weeks [61]. The study identified significantly enhanced cortical excitability and motor recovery of the upper limb in the MISAO group as compared to the MIAOO group, and concluded that MI in combination with synchronous action observation may lead to more effective neurorehabilitation in stroke patients than MI with asynchronous action observation.

Additionally, Kim et al. employed MI-based rehabilitation combined with physical training in stroke patients, and identified benefits to upper extremity motor function as measured using the FMA and the Wolf Motor Function Test [62]. Further, a study of 26 chronic stroke patients identified a benefit to upper extremity MI when used in conjunction with modified CIMT [63], and another investigation utilized MI with physical practice to enhance hand recovery [64]. Well-summarized in another review [65], studies by Page et al. indicated that MI improved function in the impaired upper limb when combined with conventional physiotherapy [66], task-oriented training [67], and CIMT [68]. Authors also noted that these beneficial changes persisted for up to three months after completion of the rehabilitation regimens. Moreover, two randomized controlled trials combining an MI protocol and conventional physiotherapy demonstrated an additive benefit to MI training [69, 70], and a 2014 meta-analysis supported the use of MI for upper extremity motor rehabilitation after stroke [71]. Taken together, these results seem to indicate a benefit to MI-based rehabilitation strategies when used in conjunction with various conventional therapies, though an optimal regimen has not yet been described.”

Although MI has become a relatively popular research topic, studies examining dose-dependence are relatively scarce. A recent investigation assessed the effectiveness of MI in 29 chronic stroke patients with mild hemiparesis by comparing MI session durations of 20, 40, and 60 minutes [72]. Subjects were administered 30-minute task-specific rehabilitation sessions 3 days/week for 10 weeks. Directly after these sessions, randomly selected subjects were administered audiotaped MI for 20, 40, or 60 minutes, while control groups were administered sham audiotapes. MI groups demonstrated reduced impairment of the affected arm (as measured by the FMA and the Action Research Arm Test (ARAT)) as compared to control groups that did not receive MI training. The experimental group exposed to MI training for 60 minutes also exhibited significantly increased FMA scores as compared to groups exposed to MI sessions of shorter duration, though no such dose-dependent response was found for the ARAT. Overall, the authors concluded that task-oriented therapies were more effective when total MI practice times were longer.

Despite the promising results presented in the preceding paragraphs, a randomized controlled trial with a larger population than prior investigations failed to uncover a therapeutic benefit to mental practice in patients within six months of stroke [73]. A 2011 Cochrane Review concluded that there exists limited evidence to suggest that MI (termed mental practice (MP) in their review) used in conjunction with other types of therapies is valuable for augmenting upper extremity function after stroke [74]. However, the group did indicate that “clinicians may consider the use of MP in addition to treatment currently used to increase upper extremity function after stroke” as “no evidence of side effects or harm was noted in the literature.”

### Lower Limb Training

The utility of MI for gait relearning has also been studied, and several randomized controlled trials have supported the use of MI for gait rehabilitation [75-78]. Additionally, a 2010 review endorsed the use of MI for retraining locomotor skills, with the caveat that it may not be effective in all patients [79]. Much like MI-based upper extremity studies, recent investigations have focused on the use of MI in conjunction with other therapeutic techniques, with the goal of maximizing recovery. An investigation of 40 hemiparetic, ambulatory stroke patients examined the effects of MI training on muscle strength and gait performance [80]. Twenty patients in the control group underwent task-oriented, lower extremity training four days/week for 45-60 minutes/day for three weeks. In addition to the task-oriented training, the experimental group (n = 20) received 30 minutes of audio-based, lower extremity training for MI practice per day. After three weeks, gait speed and four of the six muscle groups tested were significantly faster and stronger, respectively, in patients in the experimental group as compared to the control group. Further, a pilot study of 20 subacute stroke patients investigated MI in conjunction with conventional balance training [81]. The experimental group performed balance training for 20 minutes/day and an additional 10 minutes/day of MI training for three days/week for four weeks. The control group received balance training for 30 minutes/day. The preliminary evidence demonstrates that balance training along with MI may result in improved functional outcomes as compared to conventional balance training alone. Another group assessed the effectiveness of a task-oriented, circuit class training program (that included MI) for improving gait parameters in subacute stroke patients [82]. The study demonstrated that circuit training in conjunction with MI was more effective than conventional rehabilitation alone, and better promoted independent ambulation, walking speed, and endurance, among other parameters. The encouraging effect of the MI-based task-oriented training on gait abilities persisted over the six weeks of follow-up used in this study.

## Conclusions and Future Directions

Clear advantages to MI are that it is an economical, effective, non-invasive adjuvant to traditional stroke rehabilitation therapies [65]. It appears to be safe [74], and could feasibly be performed by patients at home after some instruction. Additionally, MI can be applied to each stage of stroke rehabilitation, permitting patients to begin training earlier, even in states of flaccid paralysis [83]. A number of experiments have investigated the ability of post-stroke patients to perform MI, yielding conflicting results. Not surprisingly, studies that have examined the role of MI in rehabilitation regimens for stroke patients have also generated mixed results regarding its efficacy. Future MI studies therefore, should classify patients based on specific lesion type and location in order to address the patient populations for which MI is most effective. Indeed, the results of a 2016 systematic review indicate that for patients with lesions to specific neural structures, including the frontal lobe, parietal lobe, and basal ganglia, MI may not be an appropriate rehabilitation method [27]. Further, factors including the training effects of MI, patient selection, stroke stage (acute, subacute, chronic) and the ideal manner in which to integrate MI with conventional physiotherapy techniques are critical to designing appropriate MI trials [84]. MI treatment protocols, dosage, and timing are also issues that should be considered in subsequent randomized clinical trials in order to conclusively identify the effect of MI practice in stroke rehabilitation.
